# Does higher food biodiversity in the Dutch diet lead to lower environmental impact?

**DOI:** 10.1007/s00394-026-04092-7

**Published:** 2026-09-03

**Authors:** Rosalie E. Bakker, Rosa Leek, Reina E. Vellinga, Corné van Dooren, Mary Nicolaou, Margreet R. Olthof, Ingeborg A. Brouwer

**Affiliations:** 1 Department of Health Sciences, Faculty of Science, Vrije Universiteit Amsterdam, Amsterdam, 1081 HV The Netherlands; 2 Department of Epidemiology and Data Science, Amsterdam UMC, Amsterdam, 1081 HV, The Netherlands; 3 Department of Healthy and Sustainable Nutrition, National Institute for Public Health and the Environment (RIVM), Bilthoven, 3721 MA The Netherlands; 4 WWF-NL, Zeist, 3708 JB The Netherlands; 5 Division of Human Nutrition and Health, Wageningen University and Research, Wageningen, The Netherlands; 6 Department of Public and Occupational Health, Amsterdam UMC, University of Amsterdam, Amsterdam, 1007 MB The Netherlands; 7 Amsterdam Public Health, Amsterdam UMC, 1105 AZ Amsterdam, The Netherlands

**Keywords:** Food biodiversity, Dietary species richness, Environmental impact, Life cycle assessment, Sustainable diets

## Abstract

**Purpose:**

Global diets exert substantial pressure on both public and planetary health. At the same time, diets are becoming more monotonous, with growing reliance on a limited number of staple species. Food biodiversity, the variety of biological species in the diet, and dietary environmental impact represent complementary, but distinct, dimensions of sustainable diets. However, because these dimensions capture different aspects of sustainability, it remains unclear whether diets with higher food biodiversity are also associated with lower environmental impacts.

**Methods:**

We examined associations between food biodiversity, measured as Dietary Species Richness (DSR), and six environmental impact indicators using data from the Dutch National Food Consumption Survey (DNFCS) 2012–2016. DSR scores (DSR Total, DSR Plant, DSR Animal) were calculated as the total unique species consumed over two 24-hour dietary recalls, regardless of quantities consumed. Environmental impacts associated with food consumption over the same recall period were derived from the Dutch life cycle assessment (LCA) food database (RIVM, v2019). Linear regression analyses were conducted, adjusting for age, sex, educational level, seasonality, mean daily energy intake, and weight of total food intake.

**Results:**

Higher DSR scores were generally associated with higher environmental impacts, including greenhouse gas emissions (β = 0.107, 95% CI 0.076 to 0.138), freshwater eutrophication (β = 1.28e−5, 95% CI 1.04e−5 to 1.51e−5), land use (β = 0.049, 95% CI 0.032 to 0.067), and blue water consumption (β = 0.014, 95% CI 0.012 to 0.015). Exploratory analyses adjusting for energy intake and total food intake attenuated most associations, while several associations remained statistically significant.

**Conclusion:**

Increasing DSR alone is unlikely to improve LCA-based environmental sustainability. Promoting diets that are both biodiverse and environmentally sustainable therefore requires consideration of both food biodiversity and the environmental characteristics of food production.

**Supplementary Information:**

The online version contains supplementary material available at https://doi.org/10.1007/s00394-026-04092-7.

## Introduction

Current food systems, including dietary consumption patterns, exert immense pressure on both public and planetary health [1–4]. Globally, food production and consumption account for up to 30% of total greenhouse gas emissions (GHGE), 70% of freshwater withdrawals and 78% of marine and freshwater eutrophication, placing pressure on the planetary boundaries [1, 4–6]. At the same time, agricultural intensification and specialization have contributed to biodiversity loss including an estimated 20% decline in bird populations and a 75% reduction in insect populations over recent decades [7–9].

One key driver of these trends is the transition towards intensive food production systems that prioritize high-yield crops and livestock over ecological diversity [10, 11]. This agricultural homogenization has led to a growing reliance on a limited number of staple species in global diets, diminishing both on-farm biodiversity and the variety of foods consumed [10–17]. These global trends are reflected in dietary patterns across high-income countries, such as the Netherlands, where similar shifts toward simplified, industrialized food systems have occurred. Dietary patterns in these countries are characterized by high consumption of animal-based products and ultra-processed foods (UPFs), and reliance on a limited number of crop species [10, 14, 17–19].

Food biodiversity, defined as the variety of biological species consumed in the diet, has received increasing attention in dietary intake studies [18, 20, 21]. Among the available indicators for quantifying food biodiversity, Dietary Species Richness (DSR) has been recommended as a robust metric. DSR quantifies the number of unique plant and animal species consumed in the diet, thereby capturing diversity both across and within food groups [21, 22]. Unlike traditional dietary diversity scores, which generally quantify the number of food groups or food items consumed, DSR reflects biological diversity at the species level [21, 23].

Higher DSR has been associated with improved diet quality and several health outcomes, including lower mortality risk, in European populations [24–27]. In the Netherlands, higher DSR has also been associated with better diet quality [25]. These findings are consistent with current food-based dietary guidelines, which encourage consumption of a varied diet to support human health [28]. Beyond its nutritional relevance, DSR has also been proposed as a metric that may reflect one dimension of ecological sustainability by capturing food biodiversity, which could support agricultural diversification and more resilient food systems [4, 20, 21]. As environmental sustainability is becoming increasingly integrated into European food-based dietary guidelines, this raises the question whether DSR, in addition to reflecting ecological sustainability, can also serve as an indicator of the environmental sustainability of diets.

However, food biodiversity, reflected by DSR, and environmental sustainability represent related, but distinct, dimensions of sustainable diets. In contrast, environmental sustainability is commonly evaluated using life cycle assessment (LCA)-based indicators, such as GHGE, land use, water consumption, and eutrophication, which quantify the environmental impact associated with food consumption [29–31]. These environmental impact indicators are influenced not only by the diversity of species or foods consumed, but also by the environmental characteristics of food production, including production system, geographical origin, seasonality, irrigation, storage, processing, and transport [32]. Consequently, a higher number of unique species in the diet does not necessarily translate into lower environmental impacts. It therefore remains unclear whether higher food biodiversity is associated with lower LCA-based environmental impacts [33].

To date, only one multicountry European study has examined the relationship between DSR and LCA-based environmental impacts in the European context [34]. Overall, that study reported inverse associations between plant-based DSR and both GHGE and land use in most participating countries. However, the findings were not consistent across countries. In the Dutch population, higher plant-based DSR was positively associated with both GHGE and land use, suggesting that the relationship between food biodiversity and environmental impacts may be context-specific. Furthermore, environmental sustainability encompasses multiple dimensions, and different environmental indicators may show divergent patterns [30, 32, 35–37]. Therefore, it is important to examine indicators beyond GHGE and land use to capture trade-offs and synergies between food biodiversity and environmental outcomes.

To address this, the present study examined the associations between food biodiversity, measured by DSR, and six LCA-based environmental impact indicators (GHGE, terrestrial acidification, freshwater eutrophication, marine eutrophication, land use, and blue water consumption) among Dutch adults. By evaluating multiple environmental indicators simultaneously, this study aims to provide context-specific insights into the relationship between food biodiversity and environmental impacts in the Dutch diet.

## Methods

### Study population

Data were derived from the Dutch National Food Consumption Survey (DNFCS) 2012–2016, carried out by the National Institute for Public Health and the Environment of the Netherlands [38]. DNFCS sampling is stratified to reflect the general population regarding age, sex, education, region and level of urbanization. To ensure representative samples, pregnant or lactating women and institutionalized people were not eligible for participation. Furthermore, participants needed to have an adequate understanding of the Dutch language. Besides the dietary intake data collection, every participant completed an age-specific general questionnaire, which covered various personal and lifestyle factors. A detailed description of the development and execution of this survey can be found elsewhere [38]. A total of 4313 participants' data were gathered from the DNFCS 2012–2016. For this study, only data from adults aged 19–79 years were included (N = 2078).

### Dietary intake assessment

All DNFCS 2012–2016 participants completed two non-consecutive 24-h dietary recall interviews, either by phone or face-to-face, to ensure the collection of high-quality dietary intake data. Dietary data collection was in line with guidelines from EFSA [39]. The interval between the two interviews was about four weeks and all interviews were spread equally over all days of the week and the four seasons at the population level. Data collection took place from the end of 2012 until 2016. The dietary recall interviews were led by trained dietitians who were familiar with the GloboDiet system (IARC©; former EPICSoft). GloboDiet is computer-assisted interview software that digitally stores interview information and can link it to relevant food composition data. Food consumption data were linked to the Dutch Food Composition Database (NEVO Online Version 2016/5.0) to calculate energy and nutrient intakes [40].

### Food biodiversity: dietary species richness

Dietary Species Richness (DSR) was chosen as a measure for food biodiversity in this study. DSR was previously recommended as the most appropriate measure for quantifying food biodiversity in dietary intake studies [21, 22]. In this study DSR was measured by the total count of unique species consumed over two measurement days, regardless of the quantity (calories or grams) of the consumed species. This method for calculating DSR is consistent with a previously published study using the DNFCS 2012–2016 data [25].

Dietary intake data of two 24-h dietary recall interviews were used in the calculation of participants’ DSR scores. First, all consumed species (plant, fungi or animal) within the dietary intake data needed to be identified. The raw dietary intake data consisted of food item descriptions and were matched to NEVO codes corresponding to the Dutch Food Composition Database [40]. Based on unique NEVO codes and corresponding descriptions, food items were initially classified into species according to ecological taxonomic principles (Supplementary Table S1). Several experts with ecological and botanical knowledge were consulted to check the initial species classification, especially for fruits, vegetables, pulses and lentils. NEVO codes or food registrations containing composite dishes (e.g. pre-packed frozen vegetables mix, mixed unsalted nuts etc.) were split into the different food items and corresponding species according to their recipes. The recipes, or combination of species, of these composite dishes were determined according to the information available in the national Dutch Food Composition Database. If this information was lacking, standard recipes of comparable products available in Dutch supermarkets were used to determine the included species [41]. Participants’ DSR scores were aggregated based on the sum of the total unique species consumed over two measurement days. Only unique species within participants dietary intake were counted in the final DSR scores and duplicate species were discarded regardless of differences in timing of consumption, food item variation, or quantity consumed. Three separate DSR scores were calculated, namely DSR Total, DSR Plant, and DSR Animal. DSR Plant included only plant-based species and fungi, whereas DSR Animal included only animal-based species.

DSR scores were calculated for the following food groups: fruits, vegetables, nuts and seeds, legumes, tubers, grains, meat, fish, and dairy. Alcoholic and non-alcoholic beverages, savoury snacks, added sugars, confectionery, oils, fats, condiments, spices, sauces, processed meat substitutes, and other highly processed foods were excluded. Supplementary Table S2 gives an overview of which food groups the DSR scores include and which food groups were excluded from the calculation of the DSR. Food groups or items were excluded from the DSR calculation when single species distinctions became too ambiguous. This was often the case for more processed foods, where a high variety of recipes (thus species combinations) exists, making it unclear which species to maintain. As highly processed products often comprise commonly consumed species (e.g., wheat, corn, cow, pig), we expected that excluding these products would have little impact on the count of unique species within individuals. The inclusion and exclusion of food groups in the calculation of the DSR is consistent with a previously published study that also investigated the DNFCS 2012–2016 data [25].

### Environmental impact: life cycle assessment (LCA)

Environmental impact was evaluated using the Dutch LCA food database from the National Institute for Public Health and the Environment (version 2019) and is described elsewhere [42]. The decision was made to use the first, older version of the LCA Food Database since it is more likely to align with the products and consumption patterns captured in the DNFCS 2012–2016 [43]. Environmental impact was assessed according to LCA methodology, which evaluates the impact associated with food consumption across the life cycle of food items. The scope and unit used for the environmental impact indicators were 1 kg (kg) of food consumed by the Dutch consumer and sold through a Dutch supermarket. In this study, The LCA system boundaries were cradle-to-grave and included primary production, post-harvest processing, packaging, storage and distribution, retail, preparation and consumption at home, and end-of-life processes.

The Dutch LCA food database contained primary LCA data for 242 food and drink items of the Netherlands, covering 71% of all foods consumed in the DNFCS 2012–2016. These were previously selected as the most commonly consumed food and drink items for the Netherlands. For the remaining foods and beverages included in the DNFCS, for which no primary data existed, environmental impacts were estimated through extrapolation. These mappings were performed and described previously [42]. The extrapolations were mapped by a panel of nutrition scientists using expert judgement, based on similarities in food type, origin, and food label information. All extrapolations were crosschecked by the scientific panel. Similar to the computation of the DSR scores, standard recipes were used when available in the case of composite dishes. Due to these extrapolations, environmental impact could be assessed over all food groups, in contrast to the calculation of the DSR where certain food groups had to be excluded. The environmental impact data were linked to the DNFCS and are available for the following environmental impact indicators: global warming (kg CO_2_-equivalent), terrestrial acidification (kg SO_2_-equivalent), freshwater eutrophication (kg P-equivalent), marine eutrophication (kg N-equivalent), land use (m^2^a crop equivalent (represents square meters of land occupied per year for production)) and blue water consumption (m^3^) [43]. For each participant, the environmental impact per indicator was calculated as follows: first, the LCA-derived impact per kilogram was multiplied by the reported consumption weight (kg) for each food and drink item. Subsequently, the resulting impacts were summed across the two 24-h dietary recall days. This yielded a single cumulative score per participant per indicator (e.g., GHGE in kg CO₂-eq, land use in m^2^a, blue water consumption in m^3^).

### Covariates

Demographic variables, including age, sex, and educational level were taken into account in the analyses. Educational level was derived from eight detailed educational categories and was previously aggregated into three levels: low (no education, primary education, lower vocational education, advanced elementary education), moderate (intermediate vocational education, higher secondary education), and high (higher vocational education/university).

Seasonality was also included as a potential confounder and was defined as the season in which the first 24-h dietary recall was conducted. Because dietary habits, particularly fruit and vegetable consumption, may vary across seasons, seasonal variation could influence food biodiversity and DSR scores. Although previous studies have not reported substantial confounding by seasonality in food biodiversity research, seasonality was included to facilitate comparison with earlier studies [25, 27].

Variables age and sex were also investigated as potential effect modifiers and were tested for interaction with the outcome measures.

To explore the potential influence of total food consumption, additional models were fitted that adjusted for mean daily energy intake (kilocalories/day) and weight of total food intake (grams over two days), respectively. Mean daily energy intake was calculated as the average intake of kilocalories across the two dietary recall days, whereas total food intake represented the cumulative weight (grams) of foods and beverages consumed over both recall days. These models were included because individuals with higher DSR scores may consume a greater quantity of food and therefore have higher energy intakes. In turn, both energy intake and total food consumption are associated with the environmental impacts of diets [44]. Because energy intake and weight of total food intake may also lie on the causal pathway between DSR and environmental impact, these adjustments were considered exploratory and were evaluated separately from the primary adjusted model to assess the extent to which observed associations were explained by overall food consumption.

### Statistical analysis

Frequencies and percentages were reported for categorical variables, while means and standard deviations were reported for continuous variables. Although DSR scores are considered continuous variables, DSR scores are absolute numbers of consumed species and therefore due to their discrete nature, were summarized using medians and ranges (minimum to maximum). The associations between food biodiversity (DSR Total, DSR Plant and DSR Animal) and the environmental impact associated with diets, were analyzed using linear regression models for each environmental impact indicator (global warming, terrestrial acidification, freshwater eutrophication, marine eutrophication, blue water consumption, and land use). Covariates age and sex were investigated as potential effect modifiers, however, analyses showed no significant interaction between age or sex and any of the six environmental impact indicators (p > 0.05). Beta coefficients and 95% confidence intervals were reported for the associations between DSR and the different environmental impact indicators. Besides the crude and adjusted model, two additional exploratory models were fitted. Model 1 presents the crude associations, Model 2 was adjusted for age, sex, educational level, and seasonality, and Model 3 was additionally adjusted for mean daily energy intake (kilocalories/day). To separately assess the influence of quantity of total food intake, Model 4 was adjusted for age, sex, educational level, seasonality, and weight of total food intake (g) consumed across the two dietary recall days. Survey weighting factors available within the DNFCS (e.g. demographics, season, consumption days) were not applied in the analyses since the primary objective of this study was to examine associations between DSR scores and LCA-based environmental impact indicators rather than to obtain nationally representative estimates of dietary intake or DSR. Consequently, the observed associations should be interpreted as relationships within the study population rather than nationally representative estimates for all Dutch adults. *P*-values < 0.05 were considered statistically significant. All analyses were performed in IBM SPSS Statistics Version 29.

## Results

**Fig. 1:**
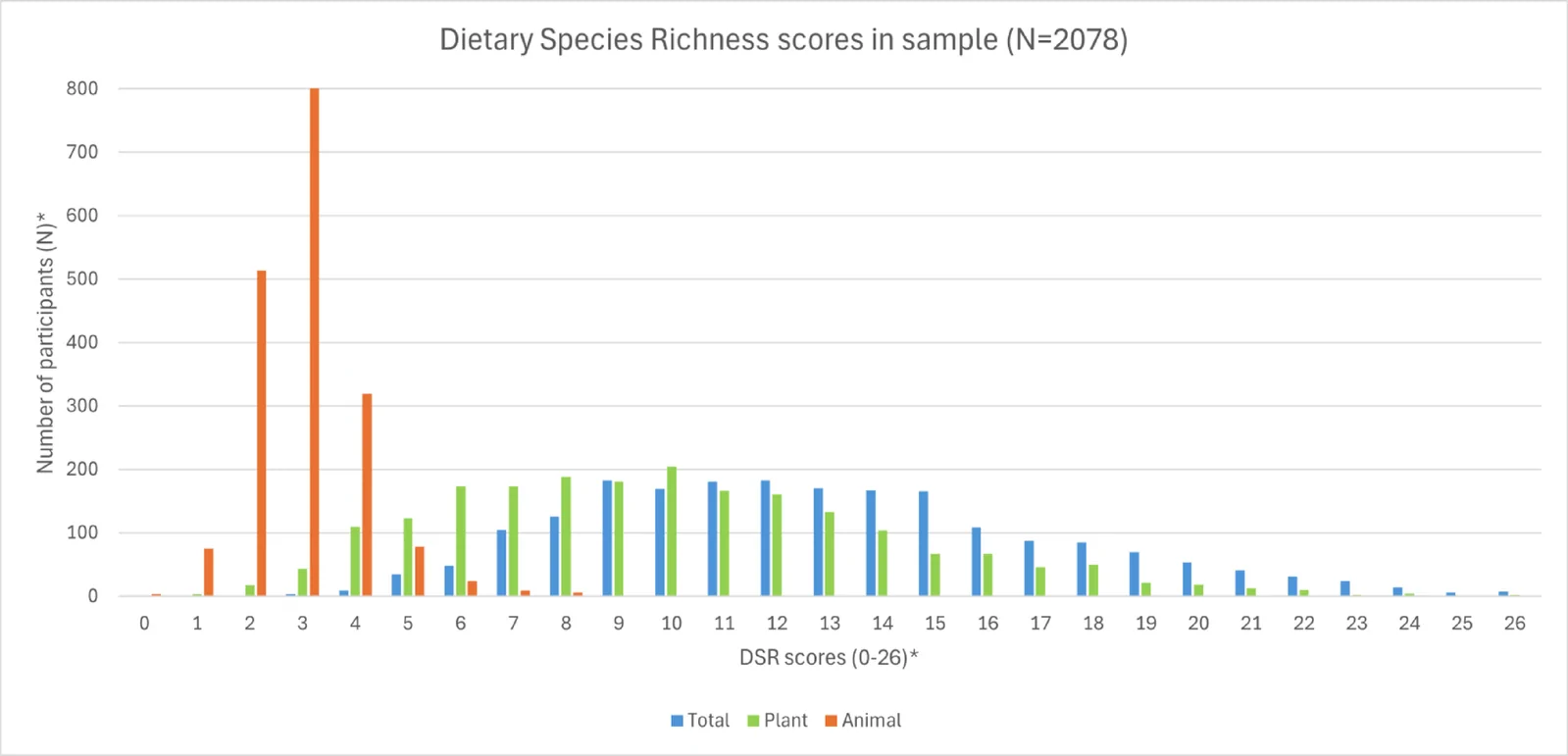
Frequency chart of Dietary Species Richness (DSR) scores over two measurement days in number of participants (N) in the Dutch National Food Consumption Survey respondents (adults aged 19–79 years): DSR Total, DSR Plant (plant-based species) and DSR Animal (animal-based species). * For readability, the Y-axis is limited to *N* = 800; the DSR Animal score for three species was in fact observed 1049 times. * For readability, the figure displays DSR scores only up to 26; scores 27–33 each had frequencies ≤3 (not displayed). DSR = dietary species richness, total count of unique species consumed over two measurement days, DSR Plant = total count of only unique plant-based species consumed over two measurement days, DSR Animal = total count of only unique animal-based species consumed over two measurement days.

This study included 2078 participants with a mean age of 50.5 years (SD 19.2) (Table 1). The majority of the participants were moderately educated (38%). Over two measurement days, the mean GHGE associated with food consumption was 10.26 kg CO₂ (SD 3.48) and the mean blue water consumption was 0.31 m^3^ (SD 0.16). Over the two measurement days, mean diet-related land use was 6.10 m^2^a crop equivalent (SD 1.99). The median DSR Total was 13 (2–33) species and the median DSR Plant was 10 species (1–28) over two measurement days. Conversely, DSR Animal had a much lower median and range over two days (3 (0–11)). Three participants in the sample did not consume any animal-based products (Fig. 1). DSR Total and DSR Plant were highly correlated (r = 0.978, *p* < 0.001).

**Table 1:** Descriptive characteristics of the Dutch National Food Consumption Survey 2012–2016 respondents, adults aged 19–79 years (*N* = 2078)

	Total (*N* = 2078)
Age in years, mean (SD)	50.5 (19.2)
Sex, *n* (%)	
Women	1043 (50.2)
Men	1035 (49.8)
Education level *n* (%)	
Low	602 (29.0)
Middle	789 (38.0)
High	687 (33.1)
Season, *n* (%)	
Spring	503 (24.2)
Summer	468 (22.5)
Autumn	484 (23.3)
Winter	623 (30.0)
Average daily energy intake in kcal, mean (SD)	2167 (690)
Total food intake in grams, mean (SD)	6520 (1769)
BMI in kg/m^2^, mean (SD)*	26.3 (5.1)
DSR Total, median (min.–max.)	13 (2–33)
DSR Plant, median (min.–max.)	10 (1–28)
DSR Animal, median (min.–max.)	3 (0–11)
Global warming in kg CO_2_ equivalent, mean (SD)	10.26 (3.48)
Terrestrial acidification in kg SO_2_ equivalent, mean (SD)	0.10 (0.046)
Freshwater eutrophication in kg P equivalent, mean (SD)	0.0007 (0.00027)
Marine eutrophication in kg N equivalent, mean (SD)	0.017 (0.008)
Land use in m^2^a crop equivalent, mean (SD)	6.10 (1.99)
Blue water consumption in m^3^, mean (SD)	0.31 (0.16)

Daily energy intake was calculated as the average kilocalories intake across two measurement days

Total food intake was calculated as the cumulative weight (grams) of foods and beverages consumed over two measurement days

DSR Total, Plant and Animal were calculated as the total count of unique species consumed over two measurement days

All six environmental impact indicators were calculated as a cumulative score per indicator, based on the reported weight of foods consumed per individual

N/n = sample size, SD = standard deviation, kcal = kilocalories, BMI = body mass index, kg = kilogram, m^2^ = square meter, CO_2_ = carbon dioxide, SO_2_ = sulfur dioxide, P = phosphorus, N = nitrogen, m^2^a = square meter per year, m^3^ = cubic meters, DSR = dietary species richness, total count of unique species consumed over two measurement days, DSR Plant= total count of only unique plant-based species consumed over two measurement days, DSR Animal = total count of only unique animal-based species consumed over two measurement days

Education level: low = no formal education, primary education, lower vocational education or advanced elementary education, middle = intermediate vocational education or higher level of general secondary education, high = higher vocational education or university degree

Season = season in which the first dietary recall day took place

*n = 1562, due to missing BMI data of participants aged 70 years and older

**Fig. 2:**
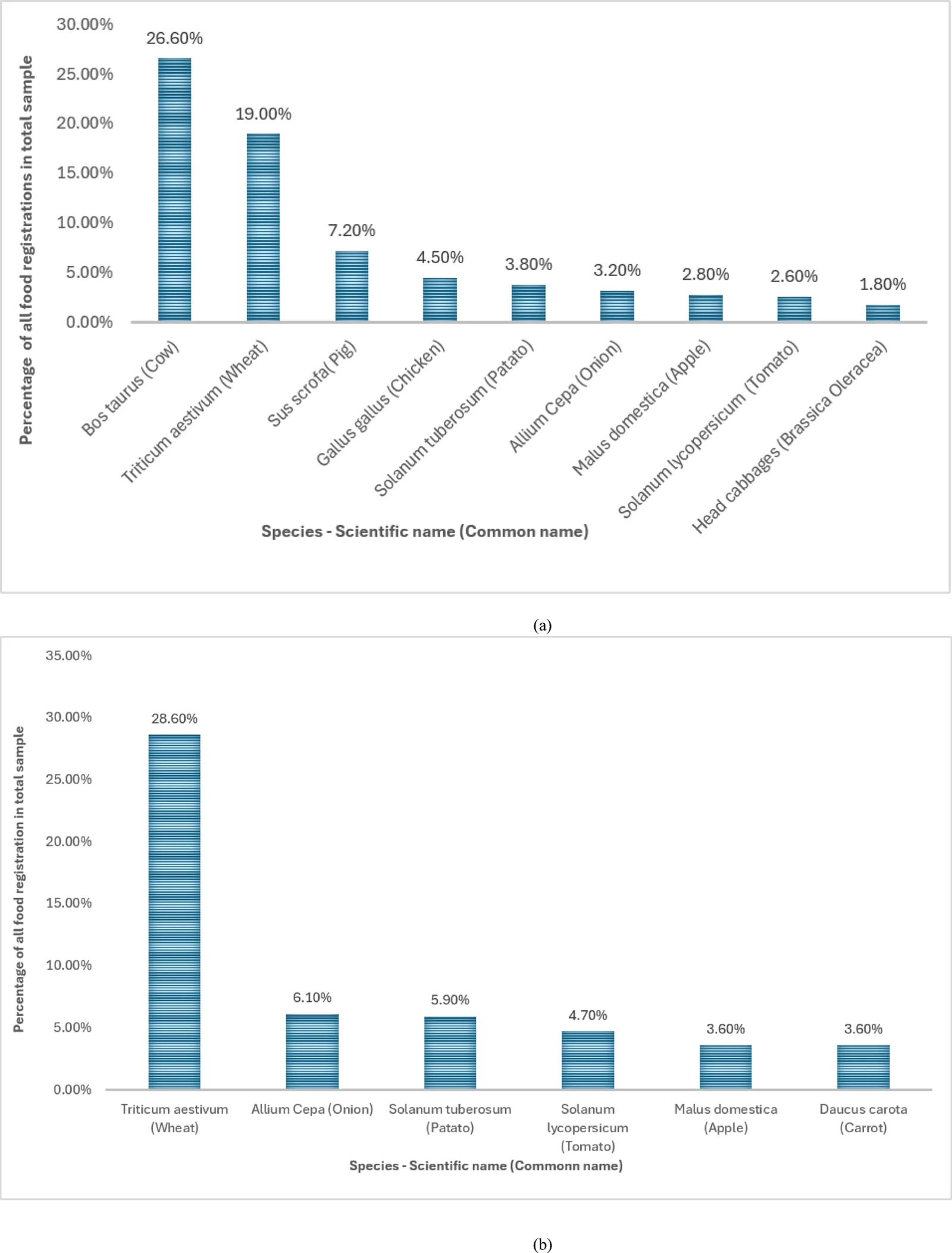
**a** Percentages of the nine most consumed species over all food registrations (n = 62,753) in Dutch National Food Consumption Survey 2012–2016, adults aged 19–79 years (N=2078). **b** Percentages of the five most consumed plant-based species over all included food registrations contributing to DSR Plant (*n* = 37,743) in Dutch National Food Consumption Survey 2012–2016, adults aged 19–79 years (*N* = 2078)

In total 157 unique species were identified in this sample. Of all included food groups, vegetables and fruits comprised the greatest number of unique species identified in this sample (Table 2). However, cow (*Bos taurus)*, wheat (*Triticum aestivum*) and pig (*Sus scrofa*) were the most consumed species over all food intake registrations over the two measurement days (Fig. 2a). Looking at the contribution of plant-based species to DSR Plant, wheat (*Triticum aestivum*), onion (*Allium Cepa*) and potato (*Solanum Tuberosum*) were the most frequently consumed species over all plant-based food registration in the sample (Fig. 2b).

**Table 2:** Number of unique species within each food group* identified within total sample

Food groups	Number of unique species within food group*
Vegetables and fungi	46
Fruits	39
Fish and shellfish	25
Nuts and seeds	17
Meat, dairy and eggs^a^	12
Grains	8
Legumes and pulses	5
Tubers	5
Total in sample	157

*Food groups and species within the classification were defined in line with national food-based dietary guidelines. Therefore, some species may be included in other food groups than in comparable international literature. For example, beans and peas are commonly considered as ‘vegetable’ in the Dutch context as opposed to legumes. An elaborate classification can be found in supplementary table S1

^a^For this table, meat, dairy, and eggs are combined into a single food group because dairy and eggs do not contribute additional unique species beyond those represented in meat

### Food biodiversity and environmental impact

**Fig. 3:**
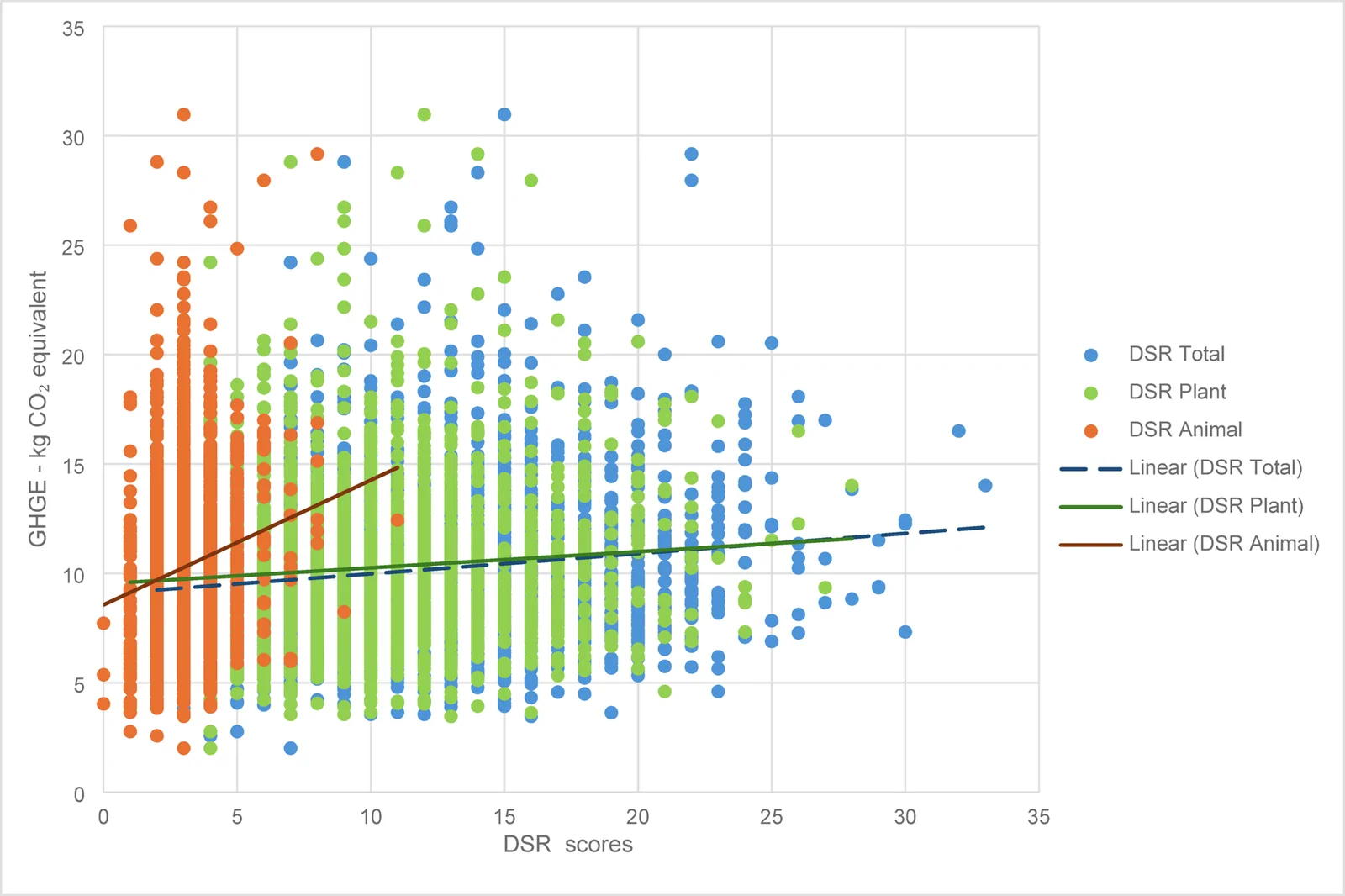
Unadjusted linear association between DSR Total, DSR Plant, and DSR Animal scores and GHGE (in kg CO₂ equivalent) in Dutch National Food Consumption Survey 2012–2016, adults aged 19–79 years (*N* = 2078)

Higher dietary species richness was generally associated with higher environmental impacts, measured in terms of GHGE, freshwater eutrophication, land use and blue water consumption (Table 3, Model 2) (Fig. 3).

**Table 3:** Associations between food biodiversity (DSR-scores) and different environmental impact indicators in the Dutch National Food Consumption Survey 2012–2016, adults aged 19–79 years (*N* = 2078)

Environmental impact indicators	DSR Total β (95% CI)				DSR Plant β (95% CI)				DSR Animal β (95%CI)			
	Model 1	Model 2	Model 3	Model 4	Model 1	Model 2	Model 3	Model 4	Model 1	Model 2	Model 3	Model 4
Greenhouse gas emissions kg CO_2_ equivalent	0.092 (0.060;0.125)**	0.107 (0.076;0.138)**	0.053 (0.028;0.077)**	0.037 (0.008;0.067)*	0.074 (0.039;0.109)**	0.094 (0.061;0.127)**	0.048 (0.022;0.073)**	0.021 (− 0.010–0.052)	0.57 (0.42;0.72)**	0.49 (0.35;0.63)**	0.21 (0.10;0.32)**	0.36 (0.23;0.49)**
Terrestrial acidification kg SO_2_ equivalent	2.55e−5 (− 4.12e−4;4.63e−4)	2.04e−5 (− 3.03e−4;0.001)	− 3.55e−4 (− 7.46e−4;3.66e−5)	− 5.04e−4 (− 9.30e−4; − 7.80e−5)*	− 4.48 (− 5.12e−4;4.22e−4)	1.56e−4 (− 3.03e−4;0.001)	− 2.80e−4 (− 6.97e−4;1.36e−4)	− 5.31e−4 (− 9.85e−4;− 7.67e−5)*	0.001 (− 0.001;0.003)	0.001 (− 0.001;0.002)	− 0.002 (− 0.004;− 4.02e−4)*	− 0.001 (− 0.003;0.001)
Freshwater eutrophication kg P equivalent	1.18e−5 (9.25e−6;1.43e– 5)**	1.28e−5 (1.04e−5;1.51e−5)**	8.43e−6 (6.63e−6;1.02e−5)**	7.59e−6 (5.34e−6;9.83e−6)**	9.00e−6 (6.30e−6;1.17e−5)**	1.04e−5 (7.83e−6;1.29e−5)**	6.66e−6 (4.73e−6;8.58e−6)**	4.83e−6 (2.43e−6;7.23e−6)**	7.96e−5 (6.85e−5;9.08e−5)**	7.32e−5 (6.28e−5;8.35e−5)**	5.13e−5 (4.34e−5;5.92e−5)**	6.32e−5 (5.37e−5;7.27e−5)**
Marine eutrophication kg N equivalent	1.15e−5 (−6.01e−5;8.31e−5)	3.42e−5 (− 3.60e−5;1.04e−4)	− 5.39e−5 (− 1.17e−4;9.40e−6)	− 8.07e−5 (− 1.50e−4; − 1.14e−5)*	9.14e−6 (− 6.73e−5;8.55e−5)	4.27e−5 (− 3.22e−5;1.18e−4)	− 3.15e−5 (− 9.89e−5;3.59e−5)	− 7.51e−5 (− 1.49e −4;− 1.24e−6)*	7.17e−5 (− 2.54e−4;3.97e−4)	− 6.56e−5 (− 3.79e−4;2.48e−4)	− 5.23e−4 (− 8.06e−4;− 2.41e−4)**	− 2.76e−4 (− 5.78e−4;2.58e−5)
Land use m^2^a crop equivalent	0.041 (0.023;0.060)**	0.049 (0.032;0.067)**	0.015 (0.002;0.028)*	0.009 (− 0.008;0.026)	0.032 (0.012;0.052)*	0.044 (0.025;0.062)**	0.014 (0.001;0.028)*	0.001 (− 0.016;0.019)	0.272 (0.187;0.357)**	0.225 (0.146;0.303)**	0.048 (− 0.009;0.106)	0.149 (0.077;0.221)**
Blue water consumption m^3^	0.015 (0.013;0.016)**	0.014 (0.012;0.015)**	0.013 (0.011;0.014)**	0.011 (0.010–0.013)**	0.016 (0.014;0.017)**	0.015 (0.013;0.016)**	0.014 (0.012;0.015)**	0.012 (0.011;0.014)**	0.019 (0.012;0.026)**	0.016 (0.009;0.023)**	0.010 (0.003;0.016)*	0.010 (0.004;0.017)*

All six environmental impact indicators were calculated as a cumulative score per indicator, based on the reported quantities of foods consumed per individual

DSR Total, Plant and Animal were calculated as the total count of unique species consumed over two measurement days

DSR = dietary species richness, total count of unique species consumed over two measurement days, DSR Plant = total count of only unique plant-based species consumed over two measurement days, DSR Animal = total count of only unique animal-based species consumed over two measurement days

β = regression coefficient, 95%CI = 95% confidence interval, kg = kilogram, CO_2_ = carbon dioxide, SO_2_ = sulfur dioxide, P = phosphorus, N = nitrogen, m^2^a = square meter years, m^3^ = cubic meters

Model 1 = crude analysis DSR and environmental impact factor

Model 2 = adjusted for age, sex, educational level (reference group = low educational level), and seasonality

Model 3 = additionally adjusted for age, sex, educational level (reference group = low educational level), seasonality, and additionally adjusted for mean daily energy intake. Daily energy intake was calculated as the average kilocalories intake over two measurement days

Model 4 = adjusted for age, sex, educational level (reference group = low educational level), and seasonality, and additionally adjusted for weight of total food intake. Total food intake was calculated as the cumulative weight (grams) of foods and beverages consumed over two measurement days

*p < 0.05

**p < 0.001

#### DSR Total

Higher DSR Total scores were associated with higher levels of GHGE (β = 0.107, 95% CI 0.076 to 0.138), freshwater eutrophication (β = 1.28e−5, 95% CI 1.04e−5 to 1.51e−5), land use (β = 0.049, 95% CI 0.032 to 0.067), and blue water consumption (β = 0.014, 95% CI 0.012 to 0.015). To illustrate, each additional species in DSR Total was associated with an approximately 0.11 kg CO_2_-eq higher GHGE**,** relative to a mean emission of 10.26 kg CO_2_-eq attributed to food consumption over two days. For blue water consumption, a one-species increase was associated with a 0.014 m^3^ (14 L) increase, compared to a mean of 0.31 m^3^ (310 L). For land use, one additional species was associated with 0.049 m^2^a higher land use over the two recall days, relative to a mean of 6.10 m^2^a attributed to food consumption over two days.

#### DSR Plant

Higher DSR Plant scores were associated with higher levels of GHGE (β = 0.094, 95% CI 0.061 to 0.127), freshwater eutrophication (β = 1.04e−5, 95% CI 7.83e−6 to 1.29e−5), land use (β = 0.044, 95% CI 0.025 to 0.062), and blue water consumption (β = 0.015, 95% CI 0.013 to 0.016). For GHGE, an increase of one plant-based species was associated with an additional emission of 0.094 kg CO₂-eq compared to a mean emission of 10.26 kg CO₂-eq attributed to food consumption over two days.

#### DSR Animal

DSR Animal was positively associated with GHGE (β = 0.49, 95% CI 0.35 to 0.63). Each additional animal-based species in the diet was associated with a 0.49 kg CO₂ eq higher GHGE, compared to a mean emission of 10.26 kg CO₂-eq attributed to food consumption over two days. For land use, a one-species increase was associated with 0.225 m^2^a higher land use over the two recall days, relative to a mean of 6.10 m^2^a attributed to food consumption over two days (β = 0.225, 95% CI 0.146 to 0.303). DSR Animal was also associated with freshwater eutrophication (β = 7.32e−5, 95% CI 6.28e−5 to 8.35e−5) and blue water consumption (β = 0.016, 95% CI 0.009 to 0.023).

#### Mean daily energy intake and total food intake

Adjustment for mean daily energy intake (Model 3) attenuated the associations between DSR and GHGE, freshwater eutrophication, land use, and blue water consumption. In addition, an inverse association was observed between DSR Animal and marine eutrophication (β = − 5.23e−4, 95% CI − 8.06e−4 to − 2.41e−4). However, these associations were very small and likely negligible in magnitude. After adjustment for energy intake, the association between DSR Animal and land use was no longer statistically significant. Overall, the magnitude of the observed associations was reduced compared with Model 2.

Adjustment for total food intake (g) instead of energy intake (Model 4) resulted in further attenuation of most associations. The associations between DSR and freshwater eutrophication and blue water consumption remained statistically significant for DSR Total, DSR Plant, and DSR Animal. In addition, the positive associations of DSR Total and DSR Animal with GHGE remained statistically significant after adjustment for total food intake (g).

In contrast, the associations of DSR Plant with GHGE and land use were no longer statistically significant after adjustment for total food intake (g). Similarly, the association between DSR Total and land use was no longer statistically significant. Significant inverse associations were observed between DSR Total and both terrestrial acidification (β =− 5.04e−4, 95% CI − 9.30e−4 to − 7.80e−5) and marine eutrophication (β = − 8.07e−5, 95% CI-1.50e−4 to − 1.14e−5), with similar patterns observed for DSR Plant. Although the previously observed associations between DSR Animal and environmental impact indicators remained after adjustment for total food intake (g), they were attenuated. Furthermore, DSR Animal became additionally inversely associated with marine eutrophication (β = − 2.76e−4, 95% CI − 5.78^e^−4 to 2.58^e^−5) after adjustment for total food intake (g).

## Discussion

This study showed that higher DSR was generally associated with higher environmental impact attributed to food consumption, including GHGE, freshwater eutrophication, land use, and blue water consumption. Although these associations were attenuated after adjustment for energy intake and, particularly, total food intake, several remained statistically significant. In contrast, inverse associations with marine eutrophication and terrestrial acidification emerged after adjustment for total food intake, highlighting that the relationship between DSR and environmental impact may differ across environmental impact indicators. These findings further suggest that greater food quantity partly explains, but does not fully account for, the observed associations.

Our findings are consistent with the Dutch results from the only previous multicountry European study examining DSR and environmental impacts, despite differences in dietary assessment methods, data granularity, and the use of older dietary intake data [34]. While that study reported predominantly inverse associations between DSR and GHGE and land use, the Dutch subsample also showed positive associations between plant-based DSR, GHGE, and land use [34]. Our study builds upon the existing evidence by confirming positive associations between DSR and environmental impact using recent high-resolution dietary intake data in the Dutch context. Previous studies focusing on dietary diversity, rather than food biodiversity per se, have similarly shown that increasing dietary diversity does not necessarily result in lower environmental impact of the diet [30, 45, 46]. For example, increased consumption of plant-based foods such as vegetables, fruits, and nuts, despite their health benefits, is associated with higher blue water consumption due to irrigation requirements [35, 47]. Another study similarly reported that although plant-based diets generally reduce GHGE and land use, they may elevate water consumption, depending on crop selection and growing conditions [32]. Moreover, cultivating a greater variety of crops can involve including species with inherently high environmental footprints and lower yields, particularly when these products are imported or produced out of season, thereby further amplifying resource demands [29].

Similar to an earlier study [27], this study found a very high correlation between DSR Total and DSR Plant, indicating that DSR in this Dutch population was almost entirely driven by plant species richness. This likely reflects the substantially larger range of plant species available and consumed within habitual diets compared with the available range of animal species. Consequently, DSR Total should largely be interpreted as reflecting plant species richness in this population, whereas DSR Animal provides complementary information on variation in animal-derived species. This also suggests that increasing DSR in similar Western populations will predominantly be achieved through greater diversity of plant foods. Importantly, although positive associations were observed for both DSR Plant and DSR Animal, the associations with GHGE and land use were stronger for DSR Animal, indicating that increasing plant species diversity while limiting animal species diversity may be more compatible with environmentally sustainable diets.

It is important to note that the environmental impact indicators included in this study represent only part of the broader concept of environmental sustainability. Although higher DSR was associated with higher LCA-based environmental impacts, these findings should not be interpreted as suggesting that lower DSR or more homogeneous diets are environmentally preferable. DSR reflects the diversity of species consumed, whereas the environmental impact indicators primarily capture impacts related to food production, resource use, and emissions. They do not account for other important aspects of biodiversity and sustainable diets, such as agrobiodiversity, soil health, wild biodiversity, genetic diversity, pesticide use, or food-system resilience. Our findings therefore suggest that increasing DSR alone is unlikely to reduce LCA-based environmental impacts; rather, both the diversity of foods consumed and the way those foods are produced should be considered when promoting sustainable diets. Although positive associations were observed for both DSR Plant and DSR Animal, the associations with GHGE and land use were consistently stronger for DSR Animal, indicating that increasing plant species diversity while limiting animal species diversity may be more compatible with environmentally sustainable diets.

Several, not mutually exclusive, mechanisms may explain the observed positive associations between DSR and environmental impacts. First, individuals consuming a greater variety of species may also consume a larger number of food items and greater total quantities of food, thereby increasing cumulative environmental burdens associated with food production, packaging, transport, storage, and food waste [32]. Although adjustment for total food intake attenuated most associations, several remained statistically significant, indicating that food quantity alone does not fully explain the findings. Second, the observed associations may primarily reflect the types of plant foods contributing to DSR Plant rather than plant species richness itself. A higher DSR Plant score may arise from greater consumption of vegetables, fruits, legumes, nuts, cereals, herbs, or imported specialty products, each of which differs considerably in environmental footprint. Moreover, even identical plant species may have substantially different environmental impacts depending on whether they are produced in open fields or heated greenhouses, grown locally or imported, consumed in or out of season, or cultivated under different irrigation and yield conditions [3, 48]. More broadly, these findings may also reflect differences between agricultural production systems. While more diverse production systems may enhance biodiversity and ecosystem resilience, they do not necessarily minimize LCA-based environmental impacts per unit of food produced, as resource-use efficiency and yields may differ between production systems [3, 11, 17, 48, 49]. Consequently, the observed associations are unlikely to reflect plant species richness alone. Finally, the so-called “vegetable paradox” may provide an additional explanation for the findings. Although vegetables and fruits are essential components of healthy diets and contribute substantially to DSR, their environmental impacts per kilocalorie may exceed those of energy-dense staple foods because larger quantities are required to provide equivalent energy intake [46, 50, 51].

This study has several strengths that enhance the robustness of our findings. By using DSR as an indicator, we were able to capture food biodiversity in a quantitative manner, allowing novel insights into the interplay between food diversity and environmental outcomes. Furthermore, the use of high-quality dietary intake data from the DNFCS 2012–2016, combined with extensive LCA data covering ‘cradle to grave’ environmental impacts, ensures a comprehensive evaluation of diet-related footprints. Since dietary recalls were used to retrieve dietary intake data, and the data were collected by trained dietitians, a lot of information about the consumed products were available, making it more attainable to derive the actual consumed species.

However, some limitations should be acknowledged. Even though high-quality dietary intake data were used in this study, potential misclassification of species cannot be fully excluded. Previous studies have estimated that between 6 and 10% of species may be misidentified in dietary intake studies, introducing bias in our results [52]. Additionally, the DSR scores used in this study did not fully capture the overall diet as we were not able to include all food categories, such as alcoholic and non-alcoholic beverages, highly processed foods, sugars, and spices, some of which may have high environmental impacts due to processing or import [53]. An additional implication of excluding certain food groups from the DSR calculation is that these foods still contributed to the environmental impact indicators. Consequently, environmental impacts attributed to excluded products, such as alcoholic beverages, sugar-containing products, and highly processed foods, were not reflected in DSR scores. This may have attenuated or obscured associations between DSR and environmental impact. However, most of these omitted food groups are associated with relatively low environmental impact compared to main food groups and also would have limited species richness, suggesting limited influence on the observed associations. Although herbs and spices would likely have increased DSR scores due to their species diversity, their environmental footprints are generally low, making substantial distortion of the association between DSR and environmental impact unlikely. Another limitation is that the DSR scores used in this study were aggregated measures. Consequently, we were unable to determine whether the observed associations were primarily driven by specific (plant) species or food groups, or differences in production systems. Moreover, such analyses would address additional research questions beyond the scope of this study. Future studies are needed to disentangle the contributions of specific plant-based species to LCA-based environmental impacts. As mentioned previously, energy intake may distort the association between DSR and environmental impact [44]. In this study, we attempted to account for this by adjusting for average daily kilocalorie intake, which resulted in attenuation of the associations. Especially in the case of DSR Animal and land use, the results show that not adjusting for mean daily energy intake would distort the association. However, the accuracy of self-reported energy intake is debatable. BMI could provide complementary information related to habitual energy balance, although it is not a direct measure of current total energy intake [54]. We did not adjust for BMI as a proxy for energy intake due to selective missing BMI values in the ≥70 years age-group (*n* missing= 516). Lastly, the environmental impact indicators depend on LCA modelling, which is necessarily based on approximations and modelling assumptions, resulting in possible under- or overestimation. This is especially relevant for supply chain components for which detailed LCA data were lacking, such as air transport in fruits and vegetables. High-resolution LCA data were available for 242 commonly consumed foods in the Netherlands, accounting for approximately 71% of total intake, while the remaining items were assigned impacts through extrapolation using market-mix assumptions [42].

Future research should further disentangle the relationship between food biodiversity and environmental sustainability by identifying which plant species or specific food groups contribute most strongly to LCA-based environmental impacts of diets. Incorporating information on production characteristics, such as seasonality, origin, greenhouse cultivation, irrigation, and transport, would substantially improve understanding of the observed associations. In addition, integrating measures of agrobiodiversity, soil health, ecosystem resilience, and wild biodiversity alongside conventional LCA indicators would provide a more comprehensive assessment of the contribution of food biodiversity to sustainable diets.

## Conclusion

In this Dutch adult population, higher DSR was generally associated with higher LCA-based environmental impacts, including greenhouse gas emissions, freshwater eutrophication, land use, and blue water consumption, although these associations were attenuated after adjustment for energy intake and particularly total food intake.

These findings suggest that higher DSR, as currently consumed, does not necessarily coincide with lower environmental impacts. However, they should not be interpreted as evidence that lower food biodiversity or more homogeneous diets are environmentally preferable, as environmental impacts depend on both dietary intake and food production-related characteristics not captured in this study.

Overall, our results do not support the use of DSR as a proxy for environmental sustainability in Dutch diets. Future dietary guidelines should therefore integrate both dietary diversity and environmental production characteristics when promoting sustainable diets.

## Supplementary Information

Below is the link to the electronic supplementary material.

Supplementary Material 1

## Data Availability

The data presented in this study are available on request from the corresponding author. DNFCS and LCA data originating from the National Public Institute of Health and Environment of the Netherlands are available for research. More information can be found on the DNFCS website: [https://www.wateetnederland.nl/](https:/www.wateetnederland.nl) and [https://www.rivm.nl/voeding/duurzaam-voedsel/database-milieubelasting-voedingsmiddelen](https:/www.rivm.nl/voeding/duurzaam-voedsel/database-milieubelasting-voedingsmiddelen) .

## References

[1] Willett W, Rockström J, Loken B et al (2019) Food in the Anthropocene: the EAT–Lancet commission on healthy diets from sustainable food systems. Lancet 393:447–492 10.1016/S0140-6736(18)31788-4 30660336

[2] Rampalli KK, Blake CE, Frongillo EA, Montoya J (2023) Why understanding food choice is crucial to transform food systems for human and planetary health. BMJ Glob Health. 10.1136/bmjgh-2022-0108766 PMC10163507 37137535

[3] Tilman D, Clark M (2014) Global diets link environmental sustainability and human health. Nature 515:518–522. 10.1038/nature13959 25383533

[4] Rockström J, Thilsted SH, Willett WC et al (2025) The EAT–Lancet commission on healthy, sustainable, and just food systems. Lancet. 10.1016/s0140-6736(25)01201-2 41046857

[5] Vermeulen SJ, Campbell BM, Ingram JSI (2012) Climate change and food systems. Annu Rev Environ Resour 37:195–222. 10.1146/annurev-environ-020411-130608

[6] Steffen W, Richardson K, Rockström J et al (2015) Planetary boundaries: guiding human development on a changing planet. Science. 10.1126/science.1259855 25592418

[7] van Strien AJ, van Swaay CAM, van Strien-van Liempt WTFH et al (2019) Over a century of data reveal more than 80% decline in butterflies in the Netherlands. Biol Conserv 234:116–122. 10.1016/j.biocon.2019.03.023

[8] Tscharntke T, Batáry P (2023) Agriculture, urbanization, climate, and forest change drive bird declines. Proc Natl Acad Sci U S A. 10.1073/pnas.2305216120 PMC10235941 37216544

[9] WWF (2024) Living Planet Report 2024 - A System in Peril. Gland, Switzerland

[10] Khoury CK, Bjorkman AD, Dempewolf H et al (2014) Increasing homogeneity in global food supplies and the implications for food security. Proc Natl Acad Sci U S A 111:4001–4006. 10.1073/pnas.1313490111 PMC3964121 24591623

[11] Khoury CK, Brush S, Costich DE et al (2021) Crop genetic erosion: understanding and responding to loss of crop diversity. New Phytol 233:84–118. 10.1111/nph.17733 34515358

[12] Monteiro CA, Moubarac JC, Levy RB et al (2017) Household availability of ultra-processed foods and obesity in nineteen European countries. Public Health Nutr 21:18–26. 10.1017/S1368980017001379 PMC10260838 28714422

[13] Foley JA, DeFries R, Asner GP et al (2005) Global consequences of land use. Science (1979). 10.1126/science.1111772 16040698

[14] Drewnowski A, Popkin BM (1997) The nutrition transition: new trends in the global diet. Nutr Rev 55:31–43. 10.1111/j.1753-4887.1997.tb01593.x 9155216

[15] Jones SK, Estrada-Carmona N, Juventia SD et al (2021) Agrobiodiversity index scores show agrobiodiversity is underutilized in national food systems. Nat Food 2:712–723. 10.1038/s43016-021-00344-3 37117466

[16] Estrada-Carmona N, Sanchez AC, Remans R, Jones SK (2022) Complex agricultural landscapes host more biodiversity than simple ones: a global meta-analysis. Proc Natl Acad Sci USA. 10.1073/pnas PMC9499564 36095174

[17] Jones AD (2017) Critical review of the emerging research evidence on agricultural biodiversity, diet diversity, and nutritional status in low- and middle-income countries. Nutr Rev 75:769–782. 10.1093/nutrit/nux040 PMC5914317 29028270

[18] FAO (2019) The state of the world’s biodiversity for food and agriculture. FAO, Rome

[19] Monteiro CA, Cannon G, Moubarac JC et al (2017) The UN decade of nutrition, the NOVA food classification and the trouble with ultra-processing. Public Health Nutr 21:5–17 10.1017/S1368980017000234 PMC10261019 28322183

[20] FAO and Biodiversity International (2017) Guidelines on assessing biodiverse foods in dietary surveys. Rome

[21] Lachat C, Raneri JE, Smith KW et al (2017) Dietary species richness as a measure of food biodiversity and nutritional quality of diets. Proc Natl Acad Sci U S A 115:127–132. 10.1073/pnas.1709194115 PMC5776793 29255049

[22] Hanley-Cook GT, Deygers J, Daly AJ et al (2025) Dietary species richness provides a comparable marker for better nutrition and health across contexts. Nat Food. 10.1038/s43016-025-01147-6 40128333

[23] Steyn N, Nel J, Nantel G et al (2006) Food variety and dietary diversity scores in children: are they good indicators of dietary adequacy? Public Health Nutr 9:644–650. 10.1079/phn2005912 16923296

[24] Huybrechts I, Chimera B, Hanley-Cook GT et al (2024) Food biodiversity and gastrointestinal cancer risk in nine European countries: analysis within a prospective cohort study. Eur J Cancer. 10.1016/j.ejca.2024.114258 39168001

[25] Bakker RE, Booij VS, van Dooren C et al (2024) Dietary biodiversity and diet quality in Dutch adults. Nutrients 16:2189. 10.3390/nu16142189 PMC11279674 39064632

[26] Hanley-Cook GT, Huybrechts I, Biessy C et al (2021) Food biodiversity and total and cause-specific mortality in 9 European countries: an analysis of a prospective cohort study. PLoS Med. 10.1371/journal.pmed.1003834 PMC8559947 34662340

[27] Aceves Martins M, Löfstedt A, Moreno-García CF et al (2025) The measurement of dietary species richness reveals that a higher consumption of dietary fibre, fish, fruits and vegetables, is associated with greater food biodiversity in UK diets. Public Health Nutr. 10.1017/S1368980025000473 PMC12100553 40211777

[28] Herforth A, Arimond M, Álvarez-Sánchez C et al (2019) A global review of food-based dietary guidelines. Adv Nutr 10:590–605 10.1093/advances/nmy130 PMC6628851 31041447

[29] Ritchie H, Rosado P, Roser M (2022) Environmental Impacts of Food Production. In: Our World in Data . https://ourworldindata.org/environmental-impacts-of-food. Accessed 12 May 2025

[30] Clark MA, Springmann M, Hill J, Tilman D (2019) Multiple health and environmental impacts of foods. Proc Natl Acad Sci U S A 116:23357–23362. 10.1073/pnas.1906908116 PMC6859310 31659030

[31] Leip A, Leach A, Musinguzi P et al (2010) Impacts of European livestock production: Nitrogen, sulphur, phosphorus and greenhouse gas emissions, land-use, water eutrophication and biodiversity. Environ Res Lett 10:115004. 10.2340/16501977-0484

[32] Poore J, Nemecek T (2018) Reducing food’s environmental impacts through producers and consumers. Science. 10.1126/science.aaq0216 29853680

[33] Baan Hofman JH, Cavero CB, Dötsch-Klerk M et al (2025) Food biodiversity and its association with diet quality and health outcomes—a scoping review. Adv Nutr. 10.1016/j.advnut.2025.100551 PMC12677162 41183784

[34] Berden J, Chimera B, Hanley-Cook GT et al (2025) Biodiverse diets present co-benefits for greenhouse gas emissions, land use, mortality rates and nutritional adequacy in Europe. Nat Food 6:857–867. 10.1038/s43016-025-01214-y 40835792

[35] Meier T, Christen O (2012) Environmental impacts of dietary recommendations and dietary styles: Germany as an example. Environ Sci Technol 47:877–888. 10.1021/es302152v 23189920

[36] Springmann M, Charles H, Godfray J et al (2016) Analysis and valuation of the health and climate change cobenefits of dietary change. Proc Natl Acad Sci USA. 10.5287/bodleian:XObxm2ebO PMC4839446 27001851

[37] Springmann M, Wiebe K, Mason-D’Croz D et al (2018) Health and nutritional aspects of sustainable diet strategies and their association with environmental impacts: a global modelling analysis with country-level detail. The Lancet Planet Health 2:451–461. 10.1016/S2542-5196(18)30206-7 PMC6182055 30318102

[38] Van Rossum CTM, Buurma-Rethans EJM, Dinnissen CS, et al (2020) The diet of the Dutch: Results of the Dutch National Food Consumption Survey 2012–2016. Bilthoven

[39] European Food Safety Authority (2009) General principles for the collection of national food consumption data in the view of a pan-European dietary survey. EFSA J. 10.2903/j.efsa.2009.1435

[40] The National Institute for Public Health and the Environment (2016) NEVO Online Version 2016/5.0. https://nevo-online.rivm.nl/. Accessed 12 Feb 2025

[41] Albert Heijn AH webshop. In: https://www.ah.nl/

[42] Vellinga RE, van de Kamp M, Toxopeus IB et al (2019) Greenhouse gas emissions and blue water use of dutch diets and its association with health. Sustainability. 10.3390/su11216027

[43] The National Institute for Public Health and the Environment (2021) LCA Food database. https://www.rivm.nl/voeding/duurzaam-voedsel/database-milieubelasting-voedingsmiddelen. Accessed 31 Jul 2025

[44] Vieux F, Darmon N, Touazi D, Soler LG (2012) Greenhouse gas emissions of self-selected individual diets in France: changing the diet structure or consuming less? Ecol Econ 75:91–101. 10.1016/j.ecolecon.2012.01.003

[45] Van Dooren C, Marinussen M, Blonk H et al (2013) Exploring dietary guidelines based on ecological and nutritional values: a comparison of six dietary patterns. Food Policy 44:36–46. 10.1016/j.foodpol.2013.11.002

[46] González-García S, Esteve-Llorens X, Moreira MT, Feijoo G (2018) Carbon footprint and nutritional quality of different human dietary choices. Sci Total Environ 644:77–94. 10.1016/j.scitotenv.2018.06.339 29981520

[47] Mekonnen MM, Hoekstra AY (2011) The green, blue and grey water footprint of crops and derived crop products. Hydrol Earth Syst Sci 15:1577–1600. 10.5194/hess-15-1577-2011

[48] Tilman D, Cassman KG, Matson PA et al (2002) Agricultural sustainability and intensive production practices. Nature. 10.1038/nature01014 12167873

[49] IPBES, Diaz S, Settele J, et al (2019) Summary for policymakers of the global assessment report on biodiversity and ecosystem services. Bonn

[50] van Dooren C, Man L, Seves M, Biesbroek S (2021) A food system approach for sustainable food-based dietary guidelines: an exploratory scenario study on Dutch animal food products. Front Nutr. 10.3389/fnut.2021.712970 PMC8440881 34540878

[51] van Dooren C, Aiking H (2016) Defining a nutritionally healthy, environmentally friendly, and culturally acceptable low lands diet. Int J Life Cycle Assess 21:688–700. 10.1007/s11367-015-1007-3

[52] Łuczaj ŁJ (2010) Plant identification credibility in ethnobotany: a closer look at Polish ethnographic studies. J Ethnobiol Ethnomed. 10.1186/1746-4269-6-36 PMC3022638 21167056

[53] Fardet A, Rock E (2020) Ultra-processed foods and food system sustainability: What are the links? Sustainability. 10.3390/SU12156280

[54] Mullaney L, O’Higgins AC, Cawley S et al (2015) An estimation of periconceptional under-reporting of dietary energy intake. J Public Health (United Kingdom) 37:728–736. 10.1093/pubmed/fdu086 25355686

