# Supplementary material for: Does higher food biodiversity in the Dutch diet lead to lower environmental impact?
Source: Eur J Nutr. 2026 Sep 3;65(7):248. doi: 10.1007/s00394-026-04092-7 (PMC13541952; doi:10.1007/s00394-026-04092-7)
Supplement: Supplementary file 1 [file 394_2026_4092_MOESM1_ESM.docx]

**Supplementary Materials**
*European Journal of Nutrition*

**Does higher food biodiversity in the Dutch diet lead to lower environmental impact?**
Rosalie E. Bakker ¹^,^⁶, Rosa Leek¹^,^⁶, Reina E. Vellinga², Corné van Dooren³^,^⁴, Mary Nicolaou ⁵^,^⁶, Margreet R. Olthof¹^,^⁶, Ingeborg A. Brouwer¹^,^⁶

¹ Vrije Universiteit Amsterdam, Department of Health Sciences, Faculty of Science, Amsterdam Public Health Research Institute, 1081 HV Amsterdam, The Netherlands
² National Institute for Public Health and the Environment (RIVM), Department of Healthy and Sustainable Nutrition, 3721 MA Bilthoven, The
³ WWF-NL, 3708 JB Zeist, The Netherlands.
⁴ Division of Human Nutrition and Health, Wageningen University & Research, Wageningen, The Netherlands
⁵ Amsterdam UMC, Department of Public and Occupational Health, University of Amsterdam, 1007 MB Amsterdam, The Netherlands.
⁶Amsterdam Public Health, Amsterdam UMC, 1105 AZ, Amsterdam

**Corresponding author:**
Rosalie E. Bakker
E-mail address: [rosalie.bakker@vu.nl](mailto:rosalie.bakker@vu.nl)

**Contents Supplementary materials:**

| Table S1 | Overview of species determined in the Dutch National Food Consumption Survey 2012-2016 respondents (adults 19-79 years). |
| --- | --- |
| Table S2 | Food items and/or food groups included in the different Dietary Species Richness (DSR) scores (overall, fruit and vegetables) in the Dutch National Food Consumption Survey 2012-2016 respondents (adults 19-79 years). |
|  |  |

**Table S1** Overview of species determined in the Dutch National Food Consumption Survey 2012-2016 respondents (adults 19-79 years).

| **Dutch common name** | **English common name** | ***Species name*** |
| --- | --- | --- |
| ***Fruit*** | | |
| Aardbei | Strawberry | *Fragaria x ananassa Duchene ex Rozier* |
| Abrikoos | Apricots | *Armeniaca vulgaris* |
| Ananas | Pineapple | *Ananas comosus* |
| Appel | Apple | *Malus domestica* |
| Banaan (excl. Bakbanaan) | Banana (excl. Plantain) | *Musa acuminata* |
| Blauwe bes | Blueberries | *Vaccinium corymbosum* |
| bosbes | Blueberries | *Vaccinium myrtillus* |
| Bramen | Blackberries | *Rubus fruticosus* |
| Citroen | Lemon | *Citrus limon* |
| Cranberry | Cranberries | *Vaccinium macrocarpon* |
| Dadel | Date | *Phoenix dactylifera* |
| Druif | Grapes | *Vitis vinifera* |
| Frambozen | Raspberries | *Rubus idaeus* |
| Goji bes | Goji berry | *Lycium barbarum* |
| Granaatappel | Pomegranate | *Punica granatum* |
| Grapefruit | Grapefruits | *Citrus paradisi* |
| Kaki | Kaki | *Diospyros kaki* |
| Kersen | Cherries (sweet and sour) | *Prunus avium* |
| Kiwi | Kiwi | *Actinidia chinensis* |
| Kokosnoot, seroendeng | Coconut | *Cocos nucifera* |
| Kruisbes | Gooseberry | *Ribes uva-crispa* |
| Limoen | Lime | *Citrus aurantifolia* |
| Lychee | Lychee | *Litchi chinensis* |
| Mandarijn | Mandarins | *Citrus reticulata* |
| Mango | Mango | *Mangifera indica* |
| Meloen (Galia, net, honing, suiker kanteloep | Melons | *Cucumis melo* |
| Moerbeien | Mulberries | *Morus (alba and nigra)* |
| Nectarine | Nectarines | *Persica vulgaris* |
| Olijf | Olives | *Olea europaea* |
| Papaja | Papayas | *Carica papaya* |
| Passievrucht | Passionfruit | *Passiflora edulis* |
| Peer | Pear | *Pyrus communis* |
| Perzik | Common peaches | *Prunus persica* |
| Pruim | Plum | *Prunus domestica* |
| Rode bes (aalbes) | Red currant | *Ribes rubrum* |
| Sinaasappel | Oranges | *Citrus sinensis* |
| Vijg | Fig | *Ficus Carica* |
| Watermeloen | Watermelon | *Citrullus lamatus* |
| Zwarte bes | Black currant | *Ribes nigrum* |
| ***Vegetables*** | | |
| Rabarber | Rhubarb | *Rheum Rhabarbarum* |
| Alfalfa | Alfalfa | *Medicago sativa* |
| Andijvie, Groenlof | Endives | *Cichorium endivia* |
| Artisjok | Globe artichoke | *Cynara cardunculus* |
| Asperges | Asparagus | *Asparagus Officinalis* |
| Avocado | Avocado | *Persea americana* |
| Aubergine | Aubergine/eggplant | *Solanum melongena* |
| Komkommer, augurk | Cucumber, gherkin | *Cucumis sativus* |
| Bamboespruiten | Bamboo | *Bambusa vulgaris* |
| Bakbanaan | Plantain | *Musa × paradisiaca* |
| Biet | Beets, chards | *Beta vulgaris* |
| Selderij (bleek) | Celeriac, celery | *Apium graveolens* |
| Bonen | Common beans | *Phaseolus Vulgaris* |
| Champignons | Cultivated mushroom | *Agaricus bisporus* |
| Chantarellen | Chanterelles | *Cantharellus cibarius* |
| Courgette, pompoen | Courgette, pompoen, squash | *Cucurbita Pepo* |
| Erwten | Peas | *Pisum Sativum* |
| Kousenband | Asparagus bean | *Vigna unguiculata* |
| Kolen | Head cabbages | *Brassica Oleracea* |
| Chinese kool, paksoi, raapstelen | Chinese cabbages, turnip | *Brassica Rapa* |
| Sopropo | Bitter melon/goya | *Momordica charantia* |
| Spaanse peper | Chili peppers | *Capsicum frutescens* |
| Koolraap | Swede | *Brassica napus* |
| Schorseneren | Black salsify | *Scorzonera hispanica* |
| Kappertjes | Caper | *Capparis spinosa* |
| Knoflook | Garlic | *Allium sativum,* |
| Paardenbloem | Dandelion | *Taraxacum officinale* |
| Paprika | Sweet bellpeppers | *Capscum annuum* |
| Pastinaak | Parsnip | *Pastinaca sativa* |
| Postelein | Common purslane | *Portulaca oleracea* |
| Prei | Leek | *Allium ampeloprasum* |
| Sla | Head lettuces | *Lactuca Sativa* |
| Mais | Maize | *Zea Mays* |
| Spinazie | *Spinach* | *Spinacia oleracea* |
| Tauge | *Bean sprouts* | *Phaseolus aureus* |
| Tuinbonen | Broad beans | *Vicia Faba* |
| Tomaat | Tomato | *Solanum lycopersicum en Lycopersicon esculentum* |
| Ui | Onions | *Allium Cepa* |
| Venkel | Fennel | *Foeniculum vulgare* |
| Witlof | Belgian Endives | *Cichorium intybus* |
| Radijs | Radish | *Raphanus sativus* |
| Waterkers | Rorippa | *Rorippa nasturtium-aquaticum* |
| Wortel | Carrots | *Daucus carota* |
| Zeekraal | Salicornia | *Salicornia (not included in DSR vegetables)* |
| Zeewier (nori) | Nori | *Porphyra umbilicalis (not included in DSR vegetables)* |
| Zeewier Kelp | Kelp | *Saccharina japonica (not included in DSR vegetables)* |
| ***Tubers*** | | |
| Aardappel (aardpeer) | Patato | *Solanum tuberosum* |
| Bataat | Sweet patato | *Ipomoea batatas* |
| Cassave, Tapioca | Cassava | *Manihot esculenta* |
| Pomtajer | Tannia | *Xanthosoma sagittifolium* |
| Yam | Yam | *Dioscorea alata* |
| ***Legumes*** | | |
| Sojaboon | Soybean | *Glycine max* |
| Bonen (black-eyed) | Beans black eyed | *Vigna radiata* |
| Mungboon | Mung beans | *vigna radiata* |
| Kikkererwt | Chickpea | *Cicer arietinum* |
| Linzen | Lentil | *Lens culinaris* |
| ***Grains*** | | |
| Haver | Oat | *Avena Sativa* |
| Boekweit | Buckwheat | *Fagopyrum Esculentum* |
| Gerst | Barley | *Hordeum Vulgare* |
| Gierst | Millet (pearl and finger) | Panicum hirticaule |
| Rijst | Asian Rice | *Oryza sativa* |
| Quinoa | Quinoa | *Chenopodium quinoa* |
| Rogge | Rye | *Secale cereale* |
| Tarwe | Wheat (common, spelt, durum, combinations) | *Triticum aestivum* |
| ***Nuts and seeds*** | | |
| Amandelen | Almonds | *Amygdalus communis* |
| Cashew noot | Cashew | *Anacardium occidentale* |
| Pinda | Peanuts | *Arachis hypogaea* |
| Paranoot | Brazil nuts | *Bertholletia Excelsa* |
| Hennepzaad | Hemp seeds | *Cannabis Sativa* |
| Pecan noot | Pecan nuts | *Carya Illinoinensis* |
| Kastanje | Chestnuts | *Castanea spp.* |
| Hazelnoot | Hazelnuts | *Corylus avellana* |
| Walnoot | Walnuts | *Juglans regia* |
| Macadamia | Macadamia | *Macadamia integrifolia* |
| Pistachios | Pistachios | *Pistacia vera* |
| Zonnebloempitten | Sunflower seeds | *Helianthus annuus* |
| Lijnzaad | Line seeds | *Linum usitatissimum* |
| Maanzaad | Poppy seeds | *Papaver somniferum* |
| Pijnboompitten | Pine nuts | *Pinus spp.* |
| Chia zaad | Chia seeds | *Salvia hispanica* |
| Sesame zaad | Sesame seeds | *Sesamum indicum* |
| ***Animal-based species*** | | |
| Koe | Cow | *Bos taurus* |
| Varken | Pig, boar | *Sus scrofa* |
| Geit | Goat | *Capra aegagrus* |
| Kalkoen | Turkey | *Meleagris gallopavo* |
| Schaap | Sheep | *Ovis aries* |
| Kip | Chicken | *Gallus gallus* |
| Eend | Duck | *Anas platyrhynchos* |
| Fazant | Pheasant | *Phasianus colchicus* |
| Struisvogel | Ostrich | *Struthio camelus* |
| Paard | Horse | *Equus ferus* |
| Konijn | Rabbit | *Oryctolagus cuniculus* |
| Hert | Red deer | *Cervus elaphus* |
| Ansjovis | Anchovies | *Engraulis encrasicolus* |
| Baars | European perch | *Perca fluviatilis* |
| Koolvis | Coalfish | *Pollachius virens* |
| Kabeljauw | Cod | *Gadus callarias* |
| Schar | Common dab | *Limanda limanda* |
| Paling | European eel | *Anguilla anguilla* |
| Krab | Edible crab | *Cancer pagurus* |
| Haring | Herrings | *Clupea harengus* |
| Heilbot | Halibut | *Hippoglossus hippoglossus* |
| Kreeft | American lobster | *Homarus americanus* |
| Schelvis | Haddock | *Melanogrammus aeglefinus* |
| Makreel | Atlantic Mackerel | *Scomber scombrus* |
| Mosselen | Common mussel | *Mytilus edulis* |
| Pangasius | Pangasius | *Pangasius pangasius* |
| Schol | European plaice | *Pleuronectes platessa* |
| Gamba | Northern prawn | *Pandalus borealis* |
| Forel | Trout (rainbow) | *Salmo gairdneri* |
| Zalm | Atlantic salmon | *Salmo salar* |
| Sardines | European sardines | *Sardina pilchardus Walbaum* |
| Garnaal | Common shrimp | *Crangon crangon* |
| Tong | Common sole | *Solea solea* |
| Inktvis | Squids | *Loligo forbesi* |
| Wijting | Whiting | *Merlangius merlangus* |
| Tilapia | Nile tilapia | *Oreochromis niloticus* |
| Tonijn | Tuna | *Thunnus alalunga* |
|  |  |  |

**Table S2** Food items and/or food groups included in the different Dietary Species Richness (DSR) scores (DSR Total, DSR Plant, DSR Animal) in the Dutch National Food Consumption Survey 2012-2016 respondents (adults 19-79 years).

|  | DSR Total | DSR Plant | DSR Animal |
| --- | --- | --- | --- |
| Included |  |  |  |
| *Food groups* | Fruits, vegetables (including fungi), nuts and seeds, legumes, tubers (including potatoes), grains, meat, fish and dairy. | Fruits, vegetables (including fungi), nuts and seeds, legumes, tubers (including potatoes) and grains. | Meat, fish and dairy. |
| *Food items* | Tofu, Tahoe and Tempeh as meat substitutes (soy bean) | Tofu, Tahoe and Tempeh as meat substitutes (soy bean) and dairy substitutes. |  |
| Excluded |  |  |  |
| *Drinks* | (Non) Alcoholic beverages (incl. coffee & tea). | (Non) Alcoholic beverages (incl. coffee & tea). | (Non) Alcoholic beverages (incl. coffee & tea). |
| *Food groups* | Savory snacks, sugar, chocolate, confectionery. | Meat, fish and dairy Savory snacks, sugar, chocolate, confectionery. Savory snacks, sugar, chocolate, confectionery. | Fruits, vegetables (including fungi), nuts and seeds, legumes, tubers (including potatoes) and grains. Savory snacks, sugar, chocolate, confectionery. |
| *Food groups* | Oils & fats. | Oils & fats. | Oils & fats. |
| *Food groups* | Condiments, spices & sauces. | Condiments, spices & sauces. | Condiments, spices & sauces. |
| *Food items* | Further processed meat substitutes. | Further processed meat substitutes. | Further processed meat substitutes. |
